# Ist das Telenotarzt-System eine sinnvolle Ergänzung im ländlichen Raum? *– Eine Analyse aus medizinischer und ökonomischer Perspektive*

**DOI:** 10.1007/s00103-022-03581-4

**Published:** 2022-09-09

**Authors:** Peter Brinkrolf, Julia Kuntosch, Bibiana Metelmann, Camilla Metelmann, Klaus Hahnenkamp, Rebekka Süss, Joachim Paul Hasebrook, Steffen Fleßa

**Affiliations:** 1 Klinik für Anästhesie, Intensiv‑, Notfall- und Schmerzmedizin, Universitätsmedizin Greifswald, Greifswald, Deutschland; 2 Lehrstuhl für Allgemeine Betriebswirtschaftslehre und Gesundheitsmanagement, Rechts- und Staatswissenschaftliche Fakultät, Universität Greifswald, Friedrich-Loeffler-Str. 70, 17489 Greifswald, Deutschland; 3 zeb business school Steinbeis Hochschule, Münster, Deutschland

**Keywords:** Rettungsdienst, Telenotarzt, Telemedizin, Innovation, Ländlicher Raum, Notfallmedizin, Emergency medical services, Tele-emergency physician, Telemedicine, Technological innovations, Rural health services, Emergency medicine

## Abstract

**Hintergrund und Ziel:**

Um die präklinische Notfallversorgung zu optimieren und aktuelle Herausforderungen zu bewältigen, wurde im Landkreis Vorpommern-Greifswald im Jahr 2017 ein Telenotarzt-System eingeführt. Es sollte aus medizinischer und ökonomischer Sicht geprüft werden, ob dies, insbesondere im ländlichen Raum, eine effiziente Ergänzung der präklinischen Notfallversorgung darstellt.

**Methodik:**

Es wurden ca. 250.000 Einsatzdaten, vor und nach Einführung des Systems, über die Jahre 2015 bis 2020 ausgewertet und ein Prä-Post-Vergleich über die Einsatzstruktur erstellt. Die 3611 Einsätze der Telenotärztinnen und -ärzte (TNA) wurden nach medizinischen Indikationen und zeitlichen Faktoren analysiert sowie mit Einsätzen ohne TNA verglichen. Zusätzlich erfolgten eine Analyse der Gesamtkosten des neuen Versorgungskonzeptes sowie eine Kostenanalyse der prä- und innerklinischen Behandlungskosten ausgewählter Erkrankungen.

**Ergebnisse:**

Das Einsatzspektrum des TNA umfasste alle Altersstufen mit verschiedenen Meldebildern, die zu 48,2 % eine mittlere Erkrankungsschwere (stationäre Behandlung erforderlich) hatten. Von Patient*innen und Mitarbeitenden wurde das System gut angenommen. Die Einsatzdaten zeigten einen signifikanten Rückgang der Notarztbeteiligung bei telenotarztfähigen Einsatzfahrzeugen um 20 %. Die jährlichen Kosten des Systems belaufen sich auf ca. 1,7 Mio. €.

**Schlussfolgerung:**

Die Ergebnisse belegen die Vorteilhaftigkeit des TNA-Systems, sodass es über die Projektdauer hinaus implementiert wurde. Das System ist medizinisch sinnvoll, funktionsfähig sowie effizient und steht als Innovation für die Umsetzung in ganz Deutschland bereit.

## Abstract (en)

**Background and objectives:**

In 2017, a tele-emergency-physician system was implemented in the county of Vorpommern-Greifswald (Germany) to optimise the prehospital emergency medical service and to counteract current challenges. It was evaluated from a medical and economic perspective whether a tele-emergency physician system is a useful addition to the existing prehospital emergency system, especially in rural regions.

**Materials and methods:**

Approximately 250,000 emergency medical service data from the years 2015 to 2020 (before and after the implementation of the telemedical system) were analysed in a pre-post comparison. A total of 3611 tele-emergency physician cases were analysed regarding medical indication and time-related factors. Additionally, total costs of the tele-emergency physician system as well as a cost analysis regarding prehospital and hospital medical costs of selected diseases were performed.

**Results:**

The tele-emergency physician treated patients of all age groups with a wide spectrum of diseases. Of the cases, 48.2% were moderate to severe but not life-threatening disorders. Patients as well as emergency medical service personnel embraced the new system. According to the data, ambulances that were equipped with the telemedical system had the number of missions requiring an emergency physician on scene reduced significantly by 20%. The yearly costs of this telemedical system amount to €1.7 million.

**Conclusions:**

The tele-emergency physician system proved to be a telemedical innovation that is medically advisable, functional and cost-efficient. Therefore, the tele-emergency physician system continued to operate after the end of the research project and is ready to be implemented across Germany.

## Einleitung

Die Aufgabe des föderal organisierten Rettungsdienstes ist die präklinische Versorgung zeitkritischer Notfallpatient*innen bei akuten Erkrankungen oder Verletzungen ebenso wie die Durchführung des qualifizierten Krankentransportes [1].

Gesellschaftliche Veränderungen und ihre Auswirkungen auf die medizinische Versorgung bedeuten für den Rettungsdienst der Bundesrepublik Deutschland erhebliche Herausforderungen, insbesondere hinsichtlich einer optimalen Verfügbarkeit und Versorgungsqualität [2]. Um kritische Patient*innen zeitgerecht zu erreichen, ist für die Planung der Notfallrettung – etwas abweichend zu anderen medizinischen Versorgungsstrukturen – die zu „versorgende Fläche“ ähnlich entscheidend wie die tatsächliche Anzahl der innerhalb dieser Fläche zu versorgenden Patient*innen.

Vor diesem Hintergrund sind ländliche Regionen mit den Herausforderungen der Organisation eines hochqualitativen Rettungsdienstes intensiver und frühzeitiger konfrontiert als Ballungsräume, sodass diesen weniger dicht besiedelten Regionen auch eine besondere Rolle in der Etablierung von Lösungen zukommt.

Trends wie die zunehmende Urbanisierung, der medizinische Fortschritt sowie der demografische Wandel verstärken sich dabei gegenseitig in ihrer Auswirkung [3]. Dies führt zu erheblichen Herausforderungen bei der Gewinnung von qualifiziertem medizinischen Personal sowie zur angemessenen Reduktion von (medizinischen) Versorgungsangeboten in vom Bevölkerungsrückgang betroffenen Regionen [4, 5]. Für den Rettungsdienst ergibt sich durch den Wegfall alternativer Versorgungsstrukturen, durch längere Transportwege sowie durch Zunahme einer älteren Bevölkerung eine gesteigerte Inanspruchnahme [6].

Auch der medizinische Fortschritt bewirkt durch eine Spezialisierung von Kliniken und Fachrichtungen längere Transportwege im Rettungsdienst; zudem kommt der präklinischen Diagnostik zur Wahl des korrekten Transportziels eine steigende Bedeutung zu [7]. Darüber hinaus ist zu erwarten, dass Vorteile in der Patientenversorgung durch selten benötigte, hochkomplexe, aber im Einzelfall lebensrettende Spezialtherapien im Rettungsdienst zunehmen werden. Die schnelle Verfügbarkeit von umfangreicher notfallmedizinischer Expertise ist somit von steigender Bedeutung, um eine gleichwertige Versorgungsqualität in unterschiedlichen Regionen zu erreichen.

Bei weiter zunehmenden Einsatzzahlen und begrenzten personellen Ressourcen – insbesondere im Bereich der notärztlichen Versorgung – ist die optimale Nutzung dieser Ressourcen essenziell. Hierzu werden verschiedene, parallele Innovationen notwendig – beispielsweise eine intensive Ausbildung der Disponenten, die über den Einsatz von Rettungsmitteln entscheiden, der Ausbau der Luftrettung, die bessere Verzahnung unterschiedlicher medizinischer Ressourcen sowie die Nutzung neuer Technologien. Durch Telemedizin könnte die (begrenzte) Ressource „Notfallmediziner*in“ effizienter genutzt werden, da dies unmittelbare Verfügbarkeit und örtliche Unabhängigkeit bedeutet sowie Anfahrtswege entfallen.

## Innovation Telenotarzt (TNA)

In telenotfallmedizinischen Systemen wird Rettungsdienstpersonal, welches sich am Einsatzort befindet, durch erfahrene Notärzt*innen von einem Computerarbeitsplatz aus begleitet. Das Ziel ist, die Qualität der Patientenversorgung zu verbessern. Durch die ubiquitäre und sofortige Verfügbarkeit notärztlicher Expertise wird das Rettungsdienstpersonal am Einsatzort unterstützt [9]. Darüber hinaus kann der TNA boden- oder luftgebundene Notärzt*innen entlasten, sodass diese für andere, potenziell kritischer erkrankte oder verletzte Patient*innen zur Verfügung stehen [10]. Der TNA kann (im Gegensatz zu den konventionellen Notärzt*innen) mehrere Einsätze parallel betreuen und Rettungswagen (RTW) in einem großen geografischen Gebiet versorgen.

### TNA-Technologie

Die Arbeit als TNA setzt hohe Expertise der Notärzt*innen voraus, daher wurde beispielsweise durch die Ärztekammern Nordrhein und Westfalen-Lippe ein Curriculum zur Qualifikation als TNA entwickelt [11]. Für die Einbindung eines TNA in den Rettungsdiensteinsatz ist sowohl die Ausstattung eines TNA-Arbeitsplatzes als auch Ausrüstung der RTW mit der TNA-Technologie erforderlich. Die erste technische Umsetzung einer solchen telemedizinischen Verbindung wurde an der Rheinisch-Westfälischen Technischen Hochschule (RWTH) Aachen entwickelt [10, 12–14]. In der umfangreichen Evaluation der Aachener Kolleg*innen wurde u. a. eine hohe Qualität der Patientenversorgung mit leitlinienadhärenter Therapie festgestellt [15–17].

Eine hohe Datensicherheit und -qualität sind essenziell. Da Notfallmedizin häufig zeitkritisch ist, muss die Verbindung in Echtzeit und ohne zeitliche Verzögerung realisiert werden. Über eine bidirektionale Audioverbindung (z. B. durch ein Headset) kann das Einsatzgeschehen geschildert und gemeinsam die weitere Diagnostik und Therapie besprochen werden. Damit der TNA die Vitalfunktionen der Patient*innen kontinuierlich überwachen kann, werden die Vitaldaten des Monitors in Echtzeit übertragen. Zusätzlich kann der TNA bei Bedarf und nach separatem Einverständnis der Patient*innen eine Videoverbindung initiieren [18].

### Indikationsgebiete

Das TNA-System ist bei Einsätzen vorteilhaft, bei denen eine ärztliche Unterstützung medizinisch sinnvoll ist, aber keine manuellen Fertigkeiten benötigt werden. Hierbei kann der TNA sowohl in der Diagnostik behilflich sein (z. B. bei Beurteilung eines 12-Kanal-EKGs), bei der Therapie (z. B. bei Delegation einer medikamentösen Therapie) als auch bei organisatorischen Aspekten (z. B. bei der Wahl eines geeigneten Zielkrankenhauses oder Abwägung einer ambulanten Therapie). Die Hilfe kann sowohl von nichtärztlichem als auch ärztlichem Rettungsdienst in Anspruch genommen werden.

Der TNA kann aus dem Einsatzgeschehen heraus zu jedem Zeitpunkt durch das Personal vor Ort kontaktiert werden. Ein weiteres Indikationsgebiet ist die Überbrückung, wenn konventionelle Notärzt*innen zwar erforderlich sind, jedoch noch auf der (längeren) Anfahrt zum Einsatzort sind. Durch den TNA können in diesen Fällen die ärztliche Diagnostik und Therapie früher begonnen werden [19].

Gerade in ländlichen Regionen kann der Weg zwischen dem Einsatzort und dem Zielkrankenhaus weit sein, was eine längere Bindung der konventionellen Notärzt*innen während des Transportes bedeutet. Auch hier kann der TNA unterstützen, indem er oder sie die Transportbegleitung übernimmt und die konventionellen Notärzt*innen wieder für andere Patient*innen alarmierbar sind. Auch bei Sekundärverlegungen zwischen 2 Krankenhäusern kann der Einsatz unter bestimmten Voraussetzungen durch einen TNA supervidiert werden.

Bei einer telemedizinischen Verbindung aus der Ferne werden auch die Grenzen der TNA-Anwendung ersichtlich. Werden manuelle Fertigkeiten (z. B. invasive Maßnahmen bei Polytraumata) benötigt, kann der TNA diese nicht übernehmen. Auch herausfordernde Ärzt*innen-Patient*innen-Gespräche, zum Beispiel bei psychiatrischen Notfällen, sind telemedizinisch nur eingeschränkt umsetzbar. Abgesehen von den medizinischen Grenzen können auch technologische Grenzen, wie beispielsweise eine geringe Netzabdeckung, eine TNA-Anwendung limitieren.

Dieser Artikel soll die Einführung der Innovation TNA im Landkreis Vorpommern-Greifswald (V-G) – einer der größten und am dünnsten besiedelten Regionen der Bundesrepublik – darstellen, empirische Evidenz aus medizinischer und ökonomischer Perspektive bieten sowie Nutzen und Grenzen der Innovation diskutieren [8].

## Methoden

Im Rahmen des Projekts Land|Rettung, das vom Innovationsfonds des Gemeinsamen Bundesausschusses gefördert wurde, wurden ab Oktober 2017 sukzessive 6 RTW im Landkreis V‑G telemedizinisch ausgestattet [20]. Von diesen waren 4 in verschiedenen Regionen ohne Notarztstützpunkt stationiert und 2 in der Stadt Greifswald, die über 2 Notarztstützpunkte verfügt. Im Landkreis V‑G waren zum Zeitpunkt der TNA-Einführung 27 RTW und 12 Notarzteinsatzfahrzeuge (NEF) im Regelrettungsdienst eingesetzt [21]. Im Rahmen eines Prä-Post-Vergleichs vor und nach Einführung des TNA-Systems wurden von Oktober 2015 bis März 2020 ca. 250.000 Einsatzdaten ausgewertet. Bis zum Abschluss der Projektevaluation war der TNA aus Greifswald bei insgesamt 3611 Einsätzen beteiligt. Diese Einsätze wurden nach medizinischen Indikationen und zeitlichen Faktoren analysiert, um die Auswirkungen zu untersuchen.

Die Methoden der unterschiedlichen Analysen werden zur besseren Übersicht jeweils im Ergebnisteil der einzelnen Aspekte erläutert.

Nach Projektende wurde der TNA im Landkreis in die Regelversorgung implementiert; weitere Regionen wurden angeschlossen. Bis April 2022 wurde der TNA in fast 7500 Fällen konsultiert.

## Ergebnisse

### Medizinische Evaluation

#### Beschreibung des Patientenkollektivs

Der Rettungsdienst versorgt Patient*innen aller Altersstufen, wobei pädiatrische Notfälle beim TNA seltener vorkommen als beim bodengebundenen Notarzt. Der TNA kann bei Patient*innen verschiedener Verletzungs- und Erkrankungsschwere kontaktiert werden. Im Erhebungszeitraum wies der TNA die Patient*innen zu 48,2 % der Kategorie „keine Lebensgefahr, aber stationäre Behandlung erforderlich“ zu. Bei 30,3 % wurde die Schwere als „gering“ eingeschätzt und nur 10,9 % der Patient*innen waren in einem lebensbedrohlichen Zustand. In ca. 10 % der Fälle wurde der Schweregrad nicht erfasst. Die Verteilung der Verletzungs- und Erkrankungsschwere spiegelt die Rolle des TNA als Bindeglied zwischen der Versorgung durch nichtärztliches Rettungsdienstpersonal einerseits und konventionellem Notarzt andererseits wider.

Ein häufiger Grund für die Kontaktierung des TNA waren Patient*innen mit starken Schmerzen (9 % der TNA-Einsätze). Dies wird ebenfalls deutlich bei der Betrachtung der Häufigkeitsverteilung der Meldebilder. In einer Subanalyse wurden 15.353 Einsätze mit bodengebundenen Notärzt*innen hinsichtlich zuvor kategorisierter Meldebilder mit 1465 TNA-Einsätzen verglichen (Tab. 1; [22]). Der Anteil an Einsätzen mit abdominellen und traumatischen Beschwerden war beim TNA signifikant höher. Bei diesen Meldebildern ist häufig eine Schmerztherapie nötig. Bei Einsätzen mit einem hohen Bedarf an manuellen Fertigkeiten (z. B. Bewusstlosigkeit) und solchen, bei denen das ärztliche Gespräch im Mittelpunkt steht (z. B. psychiatrische Einsätze), wird der TNA erwartungsgemäß seltener genutzt.

**Table 1:** 

Meldebild	Bodengebundene/r Notärzt*in (%; *n* = 15.353)	TNA (%; *n* = 1465)	Signifikanz (χ^2^-Test)
Abdominell	4,4	11	*p* < 0,001
Kardiologisch	12,2	7,7	*p* < 0,001
Weitere internistische Beschwerden	36,2	32,7	*p* = 0,007
Bewusstlosigkeit	8,8	2,2	*p* < 0,001
Neurologisch	11,9	12,3	*p* = 0,645
Psychiatrisch	6,7	0,6	*p* < 0,001
Gynäkologisch	0,6	0,1	*p* = 0,004
Pädiatrisch	0,8	0,1	*p* < 0,001
Trauma und Verletzung	9,9	15,4	*p* < 0,001
Sonstige Einsätze	8,5	18	*p* < 0,001

Ein ähnliches Verteilungsmuster konnte auch im TNA-Projekt Straubing in Bayern identifiziert werden [23]. Die häufigsten Einsatzdiagnosen in jener Modellregion waren mit je 8 % aller Primäreinsätze „traumatische Verletzungen“, „akutes Koronarsyndrom“ und „hypertensive Entgleisungen“ und mit je 7 % „akutes Abdomen“ und „Apoplex“.

In 55 % aller Primäreinsätze im Landkreis V‑G wurde durch den TNA eine Medikamentengabe delegiert. Bei 24 % der Patient*innen waren eine telemedizinisch supervidierte Diagnostik und Therapie vor Ort ausreichend, sodass auf einen Transport in ein Krankenhaus verzichtet werden konnte. Dies führt sowohl zu einer Entlastung der Notaufnahmen als auch zu einer höheren Zufriedenheit der Patient*innen.

#### Patientenzufriedenheit

In einer Befragungsstudie von 3814 Patient*innen, die vom Rettungsdienst im Landkreis V‑G von Januar bis September 2019 versorgt wurden, wurde die Zufriedenheit mit der rettungsdienstlichen Versorgung erhoben. Hierbei konnten die Antworten von 360 telemedizinisch und 1342 nichttelemedizinisch versorgten Patient*innen analysiert werden. Von den TNA-Patient*innen gaben 87 % eine volle Zustimmung zu der Aussage: „Zusammenfassend war ich mit der Betreuung und Versorgung im Rettungseinsatz zufrieden.“ Bei den Patient*innen ohne TNA-Kontakt stimmten 84 % voll zu [24]. Damit ist die Zufriedenheit der Patient*innen mit der telenotärztlichen Versorgung hoch und einer Versorgung durch den klassischen Rettungsdienst nicht unterlegen.

### Gesundheitsökonomische Evaluation

#### Kosten

Das TNA-System ist effizient, wenn seine Kosten in einem angemessenen Verhältnis zu seinem Nutzen stehen, d. h., die Bewertungsgrundlage der Vorteilhaftigkeit dieser Innovation ist die Kenntnis der materiellen sowie personellen Ressourcen [18, 25]. Tab. 2 stellt die anfallenden Kostenpositionen bei einer flächendeckenden TNA-Anwendung im Regelbetrieb der Notfallrettung dar.

**Table 2:** 

Kostenposition	Definition
**Einmalige Kosten**	
*Investitionen*	
Ausstattung pro TNA-RTW und Ausstattung TNA-Ersatz-RTW	Kosten für das telemedizinische Equipment eines RTW
Unterbrechungsfreie Stromversorgung des TNA-Arbeitsplatzes	Gewährleistung des 24/7-TNA-Regelbetriebs
Redundanz-TNA-Arbeitsplatz	Mobiliar und Hardware für TNA-Ersatz-Arbeitsplatz
*Sachkosten*	
TNA-RTW	Einbaukosten des telemedizinischen Equipments in einen RTW
Redundanz-TNA-Arbeitsplatz	Einrichtung des TNA-Ersatz-Arbeitsplatzes, für Notfälle und Schulungszwecke
TNA-Schulung	Konzeptionierung und Vorbereitung der landkreisinternen TNA-Schulung
**Jährliche Kosten**	
*Personalkosten*	
Ärztlicher Dienst	TNA und Supervisor
Verwaltung	Koordination und Verantwortung im Verwaltungsbereich
*Betriebskosten*	
TNA-RTW	Technischer Betrieb des telemedizinischen Equipments der TNA-RTW inkl. Support und Wartung
Kommunikationseinheit „PeeqBox“	Leasingkosten (für telemedizinisches Ersatzequipment)
TNA-Arbeitsplatz	Technischer Betrieb des TNA-Arbeitsplatzes
Serverinfrastruktur	Technischer Betrieb der Netzwerkinfrastruktur/Cloud (für 20–50 Rettungsmittel)
Qualitätsmanagement	Betreiberpauschale für TNA-System
Service-Techniker des Drittanbieters	Pauschale für Serviceleistung bei Störungen/Defekten des Systems
Schulungskosten	Interne und externe Schulungen und Fortbildungen des gesamten Personals

*TNA* Telenotarzt, *RTW* Rettungswagen

Insbesondere die Einführungsphase der Innovation ist durch hohe Sachkosten (Erst- und Ersatzbeschaffung nach Abschreibung) geprägt. Die telemedizinische Technik eines RTW sowie die Einrichtung des TNA-Arbeitsplatzes sind elementar. Für die Umrüstung eines RTW fallen durchschnittlich 28.500 € an. Die Anzahl der TNA-Arbeitsplätze ist abhängig vom Einsatzaufkommen und der Auslastungsrate. Die unterbrechungsfreie Stromversorgung, die Einrichtung eines Arbeitsplatzes sowie Schulungsarbeitsplatzes und die Konzeptionierung der TNA-Schulung belaufen sich auf 246.000 € [18].

Lohnzahlungen sowie fachspezifische Schulungen aller Berufsgruppen sind mit jährlich ca. 700.000 € zu beziffern, wobei Lohnsteigerungen und Personalfluktuation diesen Wert perspektivisch erhöhen. Zusätzlich sind jährlich verschiedene Betreiberpauschalen in Höhe von ca. 900.000 € für die Nutzung des Systems fällig (TNA-RTW, Arbeitsplatz, Serverinfrastruktur, Wartung und Service).

Bei einer kalkulatorischen Ausweitung des Systems auf den Landkreis V‑G wurde ersichtlich, dass die durchschnittlichen Gesamtkosten des Systems mit zunehmender Anzahl an TNA-RTW sinken. Obwohl nicht alle Kosten von der TNA-RTW-Anzahl abhängig sind, zeigt die Fixkostendegression dennoch, dass sich die systembedingt hohen Fixkosten insgesamt günstig auf die Anzahl der RTW verteilen. Zusätzlich zeigte sich, dass der TNA noch freie Kapazitäten aufweist und mehr Einsatzfahrzeuge betreuen könnte [18].

#### Entwicklung der prä- und innerklinischen Behandlungskosten

Zur ganzheitlichen Beurteilung des TNA-Systems sollen zu den Kosten der Implementierung und Anwendung weiterhin die Kosten herangezogen werden, die für die medizinische Behandlung in der prä- und innerklinischen Versorgung entstehen. Um Aussagen über mögliche Einflüsse der TNA-Anwendung auf die präklinische Versorgung und die Folgekosten in der klinischen Behandlung zu treffen, wurde eine Erhebung durchgeführt. Patient*innen, die im Zeitraum vom 01.01.2017 bis 31.12.2018 vom Rettungsdienst versorgt wurden und bei denen die Verdachtsdiagnosen „akutes Koronarsyndrom“ oder „akuter Schlaganfall“ gestellt wurden, wurden postalisch hinsichtlich ihres Outcomes befragt. Zusätzlich konnten Kostendaten aus den behandelnden Zielkrankenhäusern erhalten werden. Sekundärdaten aus 2017 (rettungsdienstliche Versorgung im Status quo) und 2018 (rettungsdienstliche Versorgung ergänzt durch das TNA-System) wurden zu einer weiteren ökonomischen Evaluation herangezogen.

Insgesamt lagen 220 Patienteneinwilligungen zur (Kosten‑)Datenanfrage an die behandelnden Krankenhäuser vor, von allen wurden durch die entsprechenden Kliniken patientenindividuelle Kostendatensätze zur Verfügung gestellt. Aufgrund von falsch-positiven (Verdachts‑)Diagnosestellungen wurden 42 Datensätze (19,09 %) verworfen. Nach der Datenvalidierung für das Jahr 2017 lagen 41 Datensätze von Patient*innen mit einem akuten Koronarsyndrom (ICD-10 I20–I25, I46) und 49 von Patient*innen mit einem akuten Schlaganfall (ICD-10 I60–I65, G45) vor. Für das Jahr 2018 waren dies 32 bzw. 56 Patient*innen.

Die Ergebnisse zu den prä- und innerklinischen Behandlungskosten werden im Folgenden kumuliert dargestellt. Alle Kosten sind auf das Jahr 2020 preisbereinigt. Die rettungsdienstlichen Versorgungskosten werden über Benutzungsentgelte (Entgelt je disponierten Rettungsmittel) gegenüber den Sozialversicherungsträgern abgerechnet. Für RTW mit TNA-Anbindung und jene ohne diese Möglichkeit wurde das gleiche Benutzungsentgelt abgerechnet.

**Figure 1:**
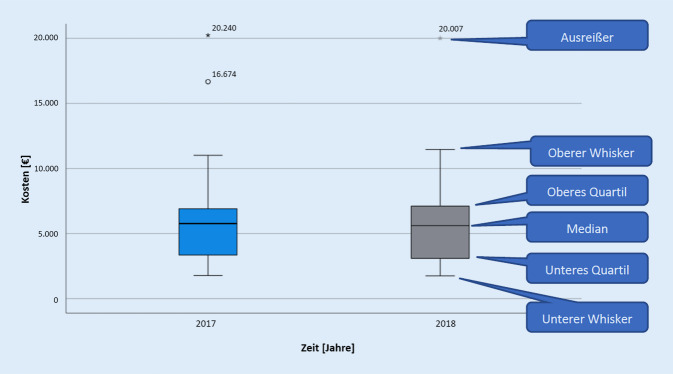

Abb. 1 gibt einen Einblick in die Behandlungskosten der Patient*innen mit einem akuten Koronarsyndrom. Die Whiskers wurden mit dem 1,5-Fachen des Interquartilsabstands berechnet und diese sich ergebende potenziell maximale Länge nach oben und unterführend an der Box ergänzt. Wenn die Kostendaten bereits innerhalb dieser errechneten Länge ihr Maximum oder Minimum erreicht haben, wird der Whisker nur bis zu diesem Wert abgetragen. Um die Übersichtlichkeit zu gewährleisten, wurde von dem Aufzeigen dreier Ausreißer (Maximum: 69.795 € im Jahr 2018) abgesehen.

Der Median ist von 2017 zu 2018 von 5770 € auf 5615 € gesunken, die Standardabweichung steigt allerdings von 6062 € auf 13.425 €. Die Disposition der Rettungsmittel unterscheidet sich in den beiden Betrachtungsjahren kaum voneinander. Nach Subtraktion der präklinischen Versorgungskosten verbleibt für 2017 ein Median der innerklinischen Behandlungskosten von 4726 € und für das Folgejahr 4590 €. Die arithmetischen Mittel der innerklinischen Behandlungskosten betragen 5667 € (2017) bzw. 8204 € (2018). Von den zur Kostenstudie eingeschlossenen 32 Patient*innen wurden 3 Personen mit dem TNA erstversorgt. Das arithmetische Mittel der innerklinischen Behandlungskosten dieser 3 Fälle beläuft sich auf 3809 € und liegt somit deutlich unterhalb des Wertes von den Patient*innen, bei denen der TNA nicht zu der Behandlung hinzugezogen wurde (8659 €). Trotz der Unterschiede bei den arithmetischen Mitteln kann von keinem signifikanten Zusammenhang zwischen TNA-Anwendung und innerklinischen Behandlungskosten ausgegangen werden (Cramers V = 0,840 mit *p* = 0,163).

**Figure 2:**
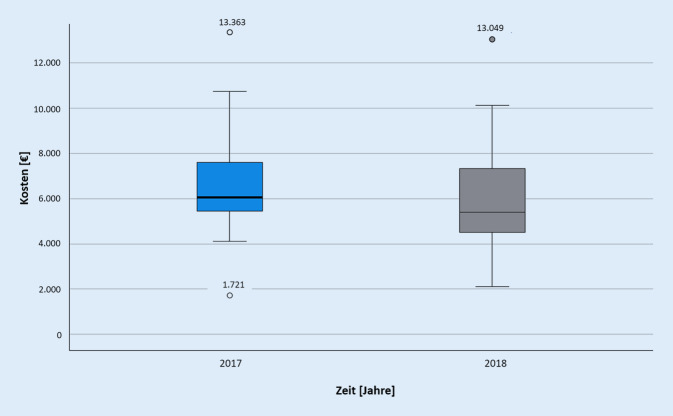

In Abb. 2 sind die Behandlungskosten der Patientenversorgung des akuten Schlaganfalls aufgeführt. Bei der Berechnung und Darstellung der Whiskers wurde gleichartig wie bei Abb. 1 vorgegangen. Auch hier wurde von der Darstellung von drei Ausreißern (Maximum: 37.028 € im Jahr 2018) abgesehen.

Im Gegensatz zu der Kostenentwicklung bei akutem Koronarsyndrom ist bei akutem Schlaganfall ein deutlicher Unterschied hinsichtlich der Verringerung des Medians ersichtlich: Dieser beträgt 2017 6060 € und 2018 5392 €. Die Standardabweichungen liegen für 2017 bei 2424 € und für 2018 bei 5350 €. Werden ausschließlich die Kosten betrachtet, die in dem Krankenhaus angefallen sind, verbleiben arithmetische Mittel in Höhe von 5744 € für 2017 bzw. 5750 € für 2018. 2018 sind 4 von 56 Patient*innen mithilfe des TNA-Systems versorgt worden. Bei deren innerklinischen Behandlungskosten liegt ein arithmetisches Mittel von 5132 € vor. Im Vergleich zum arithmetischen Mittel der Kosten der Teilnehmenden mit der Standardversorgung von 5798 € ist der Wert der TNA-Patient*innen ebenfalls niedriger. Das Cramers V zwischen der Art der disponierten Rettungsmittel (TNA-fähig bzw. nicht TNA-fähig) und den Krankenhauskosten liegt bei 0,57, was grundsätzlich auf einen starken Zusammenhang hindeutet. Dieser kann jedoch durch einen *p*-Wert des Cramers V von 0,252 nicht als repräsentativ angesehen werden.

#### Einsatzentwicklung

Um die Veränderungen der Einsatzstruktur durch die Einführung des TNA-Systems evaluieren zu können, wurde eine Prä-Post-Datenanalyse der Einsätze von RTW, NEF und TNA durchgeführt. Die Stichprobe erstreckt sich über einen Zeitraum von Januar 2015 bis Februar 2020 mit 248.279 bereinigten Datensätzen. Die Rohdaten wurden zunächst auf Plausibilität geprüft und anschließend deskriptiv ausgewertet. Da die Modellregion sukzessive mit der TNA-Technologie ausgestattet wurde, wurde dieser sechsmonatige Übergangszeitraum in der Prä-Post-Analyse nicht berücksichtigt.

Entgegen dem aktuellen Trend einer erhöhten Einsatzdisponierung, welche im Prä-Zeitraum ersichtlich war, ist der Anteil der NEF-Einsätze mit Einführung des TNA-Systems im gesamten Landkreis signifikant um 7 % (*p* = 0,024) gesunken. Der Rückgang der Einsätze mit Notarzt betrug bei den TNA-RTW 21,1 % [22]. Die zeitgleich steigenden Zahlen der TNA-Konsultationen deuten darauf hin, dass der TNA nicht erforderliche Notarzteinsätze der bodengebundenen Notärzt*innen übernehmen kann.

Die Hilfsfrist definiert die Zeit von der Alarmierung des Rettungsmittels bis zu dessen Ankunft am Einsatzort [1]. Die mittlere Hilfsfrist eines NEF hat sich im Beobachtungszeitraum von 10,3 min auf 9,2 min verringert. Die durchschnittliche RTW-Hilfsfrist hat sich mit ca. 10 min kaum verändert.

**Figure 3:**
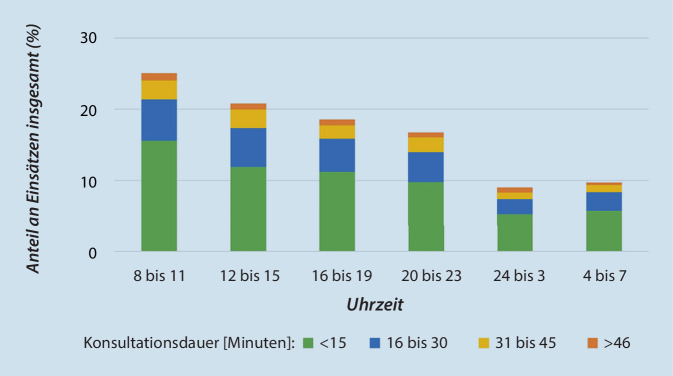

Während der Evaluationsphase betreute der TNA maximal 15 und durchschnittlich 4,4 Einsätze pro Tag, wobei der Wochentag keinen Einfluss zeigte. Knapp 28 % aller TNA-Einsätze (und damit der höchste Anteil) fanden zwischen 8:00 und 11:59 Uhr statt, wobei hiervon 62 % eine Konsultationsdauer von weniger als 15 min aufwiesen (Abb. 3). In 124 Fällen betreute der TNA 2 Einsätze parallel, wobei vormittags mehr Einsätze parallel angenommen wurden als nachmittags und abends. Der TNA entscheidet dabei fallindividuell, ob weitere Einsätze parallel betreut werden können.

Durchschnittlich war der TNA 17 min im Einsatz gebunden, wobei 50 % der Einsätze nicht länger als 11 min andauerten. Die längste beobachtete Einsatzzeit aller Einsätze (auch bei Sekundärverlegungen) betrug 3 h. Allerdings ist hier zu bedenken, dass auch Fehldokumentationen durch verspätetes Schließen eines Einsatzes nicht ausgeschlossen werden können. Wurde während eines Einsatzes klar, dass der TNA nicht benötigt wurde, war er im Mittel lediglich 4 min gebunden.

Eine weitere Aufgabe des TNA ist die Betreuung der Sekundärverlegungen, d. h. des Patiententransportes zwischen 2 Krankenhäusern. Die Notarztbeteiligung bei Sekundärverlegungen hat sich in der Prä-Post-Betrachtung von 15,2 % auf 7,5 % halbiert, wobei die TNA-Beteiligung vergleichsweise gering ausfällt. Hier bestünde zukünftig noch Potenzial, die Vorteile der telenotärztlichen Versorgung zu nutzen. Daher wurde der Standort eines TNA-RTWs verlegt und befindet sich nun an einem Krankenhaus, das häufig Sekundärverlegungen durchführt.

## Diskussion

Im Rahmen des Projektes Land|Rettung konnte das TNA-System erfolgreich im ländlichen Raum implementiert werden, nachdem es bereits seit 2011 in der Stadt Aachen regelhaft mit gutem Erfolg zum Einsatz kommt. Viele Veröffentlichungen zeigen, dass das System von medizinischem Nutzen ist und notärztliche Ressourcen entlasten kann [15, 25, 26]. So konnten auch wir in unserer Auswertung darstellen, dass durch die Anbindung von RTW an das TNA-System die Notarztquote dieser Fahrzeuge um mehr als 20 % gesenkt werden konnte. Dieser signifikante Rückgang führt zu einer Ressourcenschonung und einer Erhöhung der Verfügbarkeit der (zumeist knappen) Ressource „Notfallmediziner*in“. Im Falle eines notärztlichen Paralleleinsatzes innerhalb eines Einsatzgebietes wird der zweite Notfall mit einer erheblich längeren Anfahrtszeit konfrontiert. Insofern ist es gerade in weniger dicht besiedelten Regionen von großer Bedeutung, qualifizierte Rettungsmittel bedarfsgerecht einzusetzen und durch die Reduktion von Einsätzen, in denen das jeweilige Rettungsmittel nicht erforderlich ist, eine möglichst hohe Verfügbarkeit der Notärzt*innen zu erreichen.

Unsere Auswertungen legen nahe, dass der TNA eine wichtige Ressource sein kann, um dieses Ziel zu erreichen. Wird keine physische Anwesenheit von Notfallmediziner*innen benötigt, kann der TNA einen Einsatz ressourcenschonender und kostengünstiger betreuen, da im ländlichen Raum lange Anfahrtswege entfallen und die Konsultationsdauer eines TNA-Einsatzes wesentlich geringer ist als die Bindungszeit konventioneller Notärzt*innen [27]. Dies wird zukünftig auch vor dem Hintergrund des bereits bestehenden und sich mutmaßlich verschärfenden Personalmangels im (not‑)ärztlichen Bereich an Relevanz gewinnen.

Gleichzeitig wurde deutlich, dass der TNA im Vergleich zu boden- oder luftgebundenen Notärzt*innen nicht alle Einsatzstichworte/-meldebilder gleichermaßen versorgt. Dies ist insbesondere in Kombination mit der Tatsache zu bewerten, dass der TNA bei Erfordernis manueller Fertigkeiten nicht geeignet ist, Notfallmediziner*innen vor Ort zu ersetzen. Besonders bei kritischen Notfallsituationen, wie beispielsweise Herz-Kreislauf-Stillständen oder schwer mehrfach verletzten Personen (sogenannte Polytraumata), ist zeitnah die physische Anwesenheit von Notärzt*innen am Einsatzort erforderlich, um das Leben der Patient*innen zu retten. Insofern sollte die Senkung der Notarztquote im Sinne einer Qualitätssteigerung durch höhere Verfügbarkeit der Ressource Notärzt*in gewertet werden und auf keinen Fall als Möglichkeit einer Reduktion von Notarztstandorten in Regionen, in denen lange Anfahrtswege vorherrschen, fehlinterpretiert werden. Eine solche Reduktion setzt voraus, dass Notfallmediziner*innen auf anderem Weg zeitnah den Einsatzort erreichen können; hier käme prinzipiell ein Ausbau der Luftrettung infrage. Allerdings ist diese derzeit auf Sichtflugbedingungen angewiesen, was die Verfügbarkeit entscheidend reduziert [7]. Sofern ein sichtunabhängiger Flug auch im Rahmen der Primärrettung möglich und vor allem gesetzlich zulässig ist, würden sich in Kombination mit der Telemedizin vermutlich erhebliche Potenziale zur Reduktion von Notarztstandorten bieten.

Mögliche finanzielle Vorteile, die sich aus einer derartigen Reduktion von Notarztstandorten ergeben würden, konnten im Rahmen dieser Arbeit nicht bewertet werden, da sie – wie skizziert – von weiteren, derzeit nicht gegebenen Faktoren abhängen. Im Rahmen dieses Artikels wurden die beispielhaften Kosten der Einführung und des Betriebs eines TNA-Systems umrissen, hierbei sei darauf hingewiesen, dass die dargestellten Kosten vom Anbieter abhängig sind und sich langfristig durch die Veränderung des Marktes anpassen. Weiterhin können standardisierte Prozesse, wie die Ausbildung der TNA, zu Kosteneinsparungen führen. Diese betragen unter Berücksichtigung von laufenden Kosten und Abschreibungen derzeit ca. 1,7 Mio. € jährlich.

Im Vergleich zu einem bodengebundenen Notarztstandort ist anzunehmen, dass die Personalkosten des TNA trotz gleicher Besetzungsdauer geringfügig größer sind, da höher qualifizierte/erfahrenere Ärzt*innen eingesetzt werden. Hinzu kommen Betriebskosten für den technischen Betrieb in nicht unerheblicher Höhe, während für einen Notarztstandort zusätzliche Personalkosten für die Rettungsassistent*innen oder Notfallsanitäter*innen, die gemeinsam mit dem Notarzt ausrücken, Absetzung für Abnutzung (AfA) für das NEF sowie Mietkosten für den Standort anfallen. Zusammenfassend kann näherungsweise geschätzt werden, dass die Kosten für den Betrieb eines TNA-Standortes höher liegen als für einen bodengebundenen konventionellen Notarztstandort, jedoch die Kosten, die für 2 Notarztstandorte anfallen, nicht überschreiten.

Mit Blick auf die Kosten des TNA-Systems konnten wir projektübergreifend darstellen, dass die Kosten bei Zentralisierung deutlich geringer ausfallen als bei einer dezentralen Lösung. Dies begründet sich vor allem darin, dass bei der dezentralen Lösung nicht nur für jeden Landkreis eine eigene TNA-Zentrale aufgebaut werden muss, sondern dass auch wesentlich mehr TNA benötigt werden, die dann eventuell nicht ausgelastet sind [28]. Ein zentralisiertes TNA-System bietet besonders im Hinblick auf die technische Vernetzung sowie deren Nutzung und bezüglich der Reduzierung von Personal großes Potenzial für Kosteneinsparungen.

Wie in Abb. 3 dargestellt, ist das Einsatzaufkommen stark uhrzeitabhängig. Ein zentralisierter Ansatz könnte diesem Aspekt besser Rechnung tragen, da in großen Regionen zur Spitzenlastabdeckung zeitweise ein zweiter Arbeitsplatz in Betrieb genommen werden könnte. Selbstredend ist das Potenzial einer bundesländerübergreifenden Vernetzung und Zentralisation der TNA-Zentralen (insbesondere bei regionaler Überlast) erheblich, erfordert jedoch eine Abwägung zwischen der Notwendigkeit regionaler Kenntnisse und den ökonomischen Bedingungen [28].

Bislang liegen in der Literatur keine vergleichbaren Analysen zu prä- und innerklinischen Behandlungskosten im Prä-Post-Vergleich vor. Wir können jedoch auf Basis der relativ beschränkten Stichprobe feststellen, dass kein signifikanter Unterschied zwischen 2017 mit der Standardversorgung und 2018 mit der zusätzlichen Anwendung des TNA bezüglich der Behandlungskosten innerhalb der einbezogenen Diagnosegruppen zu beobachten ist. Die Vorteile des TNA sowohl für die Behandlungsqualität als auch für die Kosten liegen folglich im Bereich des Rettungsdienstes, nicht in der anschließenden Krankenhausbehandlung. Für aussagekräftigere Ergebnisse bleibt zu empfehlen, das innovative TNA-System hinsichtlich der Auswirkungen für die Sozialversicherungsträger anhand einer größeren Patientengruppe ökonomisch zu evaluieren. Darüber hinaus sind mögliche Einsparpotenziale aktuell noch nicht realisiert. Zu guter Letzt unterliegen die Behandlungskosten von Patient*innen einer Reihe von Einflussfaktoren, die anhand großer Fallzahlen mittels einer multivariaten Analyse hinsichtlich ihrer Bedeutung für die Kosten untersucht werden könnten.

Auffällig ist, dass der TNA in unserer Auswertung mit 24 % der Fälle einen hohen Anteil an Patient*innen ambulant behandelt. Die angewandte Methodik der Datenerhebung und Auswertung lässt keine Rückschlüsse zu, ob die Entscheidung, die Patient*innen nicht zu transportieren, durch den TNA im Einsatzverlauf getroffen wurde oder ob der TNA durch das Rettungsdienstfachpersonal verstärkt zur rechtlichen Absicherung hinzugezogen wurde in Fällen, in denen durch das Personal vor Ort schon eine ambulante Versorgung gebahnt war. In jedem Fall wirft dieser vergleichsweise hohe Anteil weitere Fragestellungen mit ökonomischem und medizinischem Bezug auf. Insbesondere stellt sich die Frage, ob der Rettungsdienst in diesen Einsätzen erforderlich war oder ob die Versorgung durch andere Ressourcen im Gesundheitswesen hätte effizienter erfolgen können. Sofern dies der Fall wäre, sollte überprüft werden, welches Vorgehen bei der Bearbeitung von Notrufen geeignet ist, weniger kritische oder weniger dringliche Fälle möglichst trennscharf zu identifizieren, um die jeweils optimale Ressource einzusetzen.

## Fazit

Das TNA-System ist medizinisch sinnvoll, funktionsfähig, effizient und steht als Innovation bereit für die Umsetzung in ganz Deutschland.
